# From Checkerboard‐Like Sand Barriers to 3D Cu@CNF Composite Current Collectors for High‐Performance Batteries

**DOI:** 10.1002/advs.201800031

**Published:** 2018-03-27

**Authors:** Jian Luo, Wei Yuan, Shimin Huang, Bote Zhao, Yu Chen, Meilin Liu, Yong Tang

**Affiliations:** ^1^ School of Mechanical and Automotive Engineering South China University of Technology Guangzhou 510640 China; ^2^ School of Materials Science & Engineering Georgia Institute of Technology Atlanta GA 30332‐0245 USA

**Keywords:** batteries, carbon nanofibers, composite materials, current collectors, electrochemical cells

## Abstract

While the architecture, surface morphology, and electrical conductivity of current collectors may significantly affect the performance of electrochemical cells, many challenges still remain in design and cost‐effective fabrication of highly efficient current collectors for a new generation of energy storage and conversion devices. Here the findings in design and fabrication of a 3D checkerboard‐like Cu@CNF composite current collector for lithium‐ion batteries are reported. The surface of the current collector is modified with patterned grooves and amorphous carbon nanofibers, imitating the checkerboard‐like sand barriers in desert regions. Due to a combined effect of the grooves and the carbon nanofibers, a battery based on this current collector retains a reversible capacity of 410.1 mAh g^−1^ (beyond the theoretical capacity of carbonaceous materials of 372 mAh g^−1^) with good capacity retention (greater than 84.9% of the initial capacity after 50 cycles), resulting in 66.2% and 42.6% improvement in reversible capacity and capacity retention, respectively, compared to the batteries using traditional Cu current collectors. Based on the excellent electrochemical performance, this composite current collector is believed to be an attractive alternative to the traditional commercially used current collectors for the anode of high‐power energy storage systems.

## 1 Introduction

Lithium‐ion batteries (LIBs) are considered one of the most promising power sources for next‐generation electronic devices and electric vehicles.^1^, ^2^, ^3^, ^4^, ^5^, ^6^ To date, carbonaceous materials are still widely used as the anode for commercial LIBs because of their alluring features such as light weight, high conductivity, good chemical stability, and low cost.^7^, ^8^, ^9^, ^10^, ^11^, ^12^, ^13^, ^14^ With the rapid advancement in energy‐consuming devices, however, traditional carbon‐based anodes no longer satisfy the increasing demands for higher energy density because carbonaceous materials have a theoretical capacity of only 372 mAh g^−1^, which severely limits the further development of high‐capacity commercial LIBs.

To overcome the limitation of low capacity, many anode materials with larger theoretical capacity have been investigated for high‐energy LIBs, including nanostructured transition‐metal oxides,^15^, ^16^, ^17^, ^18^, ^19^, ^20^ Sn‐based materials,^21^, ^22^, ^23^, ^24^, ^25^ and Si‐based materials.^26^, ^27^, ^28^, ^29^, ^30^ Depending on the superior lithium storage properties of the advanced anode materials, the reversible capacity of LIBs can be effectively increased. However, the synthesis and processing of these advanced anode materials usually require various intricate fabrication processes and high‐cost raw materials. The above defects greatly hinder these materials from becoming the mainstream commercial anode materials of LIBs, although some of them have been applied in commercial batteries. Accordingly, it is still a grand challenge to develop effective and low‐cost strategies to enhancing battery performance.

As an indispensable component in a battery, the current collector plays an important role in carrying electrode materials and collecting the current during the discharge–charge processes. Hence, the surface structures of a current collector may greatly affect the mechanical strength of electrodes and the effectiveness of electrical current collection. However, most of the commercially used current collectors for the anodes of LIBs are made of electrolytic copper foils with either smooth or rough surfaces on both sides, which severely hinders the further development of commercial LIBs. Therefore, properly designed current collectors may contribute to the development of future LIBs with larger capacity and higher power. To this end, it is an effective strategy to design, control, and optimize the structure and surface morphologies of current collectors in order to improve the electrochemical performances of LIBs. In particular, the use of 3D metal‐31, 32, 33, 34, 35, 36 and carbon‐based^37^, ^38^, ^39^, ^40^, ^41^, ^42^ materials with porous nanostructures have paved the way to the development of new‐style current collectors. Batteries with these 3D current collectors are expected to have higher electrochemical performances due to the following structural advantages: (a) 3D porous structures increase the contact area between the electrode material and current collector, and thus reduce the effective resistance of the electrode;^33^, ^35^, ^36^ (b) 3D porous structures allow more intimate contact between the electrode material and the electrolyte, thus facilitating fast transport of electrons and Li‐ions;^37^, ^39^, ^41^ (c) nanosized structures help to shorten the diffusion path for both Li‐ions and electrons, thus effectively reducing the impedance to diffusion or mass transfer in the electrode.^38^ Nevertheless, the fabrication process of such 3D current collectors often requires hazardous chemical reactions and additional treatments, which increase the risk and cost of its manufacturing.

Considering the efficiency and cost of large‐scale production, most electrodes of commercial LIBs are currently fabricated by a low‐cost slurry coating process. In this case, hierarchical composite current collectors with special nanostructures have great potential to improve the performance of future commercial LIBs. It is believed that nanostructured current collector films with excellent lithium storage properties may not only enhance lithiation/delithiation of the electrode materials but also increase the mechanical strength of the electrode, as reported in the literature.^43^, ^44^ Thus, the performance of a battery, including reversible capacity, rate capability, and cycle life, may be greatly improved by modifying the surface structures and compositions of the hierarchical composite current collector.

Interestingly, encompassing the issues of desertification and global climate change, semiburied sand barriers with a checkerboard‐like pattern have been widely used to control the near‐surface sand flow in arid desert regions.^45^, ^46^, ^47^ Such sand barriers can halt near‐surface sand flow by increasing underlying surface roughness, reducing near‐surface wind speed, and weakening sand transportation intensity, thereby stabilizing the sand surface.^48^, ^49^ Inspired by such a sand‐fixation principle of the checkerboard‐like sand barriers, we carefully examined a 3D controllable checkerboard‐like Cu@CNF graphical composite current collector for LIBs. This checkerboard‐like composite current collector inherits the superior properties of carbon nanofibers and large surface area. Because of the combined effect of the amorphous carbon nanofibers and patterned grooves, the bonding strength between the electrode material and current collector can be significantly enhanced. Consequently, a battery based on this current collector shows a remarkable improvement in reversible capacity, rate capability, and cycle life. Moreover, a novel and efficient process for nickel plating has also been developed. Unlike the traditional electroless nickel plating process, our method does not require expensive and complex procedures of sensitization and activation. Since this checkerboard‐like composite current collector owns a series of advantages such as high electrochemical performance, cost‐effective material, and simple fabrication procedure, we believe it can be a good supplementation to demonstrate an idea for the research of future commercial LIBs and other energy storage systems.

## 2 Results and Discussion

**Figure 1:**
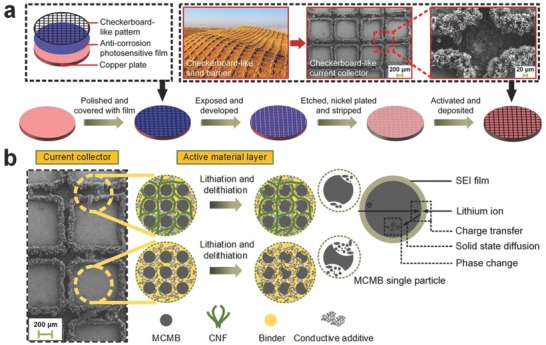
Schematic diagrams of a) preparation procedure of the checkerboard‐like Cu@CNF current collector and b) repeated discharge–charge processes of the electrode based on the checkerboard‐like Cu@CNF current collector.

**Figure 2:**
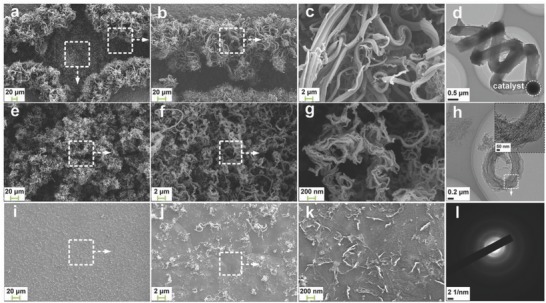
a) Top‐view morphology (SEM images) of the checkerboard‐like Cu@CNF current collector; b,c) SEM and d) TEM images of the carbon nanofibers at the edge of the convex for the checkerboard‐like Cu@CNF current collector shown in a); e–g) SEM and h) TEM images of the carbon nanofibers in the groove of the checkerboard‐like Cu@CNF current collector shown in (a); i–k) Surface morphology of the convex for the as‐prepared checkerboard‐like Cu@CNF current collector; l) SAED pattern of the selected region in (h). Inset in (h): magnified TEM image of the selected region in (h).

The formation strategy of the checkerboard‐like Cu@CNF current collector is schematically depicted in **Figure** **1**a. After the nickel plating and activation processes, the grooves on the surface of the copper plate were filled with activated catalysts. With the assistance of catalysts, amorphous carbon nanofibers were regularly deposited on the pretreated copper plate using chemical vapor deposition (CVD) method. To certify the above statement, surface structures and morphologies of the as‐prepared current collector were characterized by both scanning electron microscope (SEM) and transmission electron microscope (TEM) tests. As shown in **Figure** **2**a, the SEM image displays the surface structures of the checkerboard‐like Cu@CNF current collector. Carbon nanofibers were observed on the surface of patterned grooves. These patterned grooves conduce to increasing the surface area of the current collector and enhancing storage of the electrode materials. With increasing the surface area, the effective contact points between the electrode material and current collector can be greatly intensified. The sufficient contact points help to improve the electrical conductivity of the electrode and the bonding strength between the electrode material and current collector. Besides, the volume variation of the active material stored in the grooves can be partly restrained in the presence of patterned grooves. After the CVD procedure, carbon nanofibers were regularly deposited along the edge of the convex and in the grooves, intertwining with each other. Under the combined effect of the groove template and twining structure, the carbon nanofibers can be well preserved on the current collector. However, we can find an obvious difference in the diameter of the carbon nanofibers between these two sites. The carbon nanofibers at the edge of the convex have a larger diameter within the range of 0.5–2 µm (see Figure 2b,c), compared with those in the grooves (diameter range: 50–100 nm), as can be seen in Figure 2e–g. This is because the flow rate of the gas mixture at the edge of the convex is much faster than that in the grooves. As a result, there are more carbon atoms deposited at the edge of the convex per unit time, thereby leading to a larger diameter of the carbon nanofibers, which yield a better mechanical property. This may be beneficial to the improvement in the bonding strength between the electrode and current collector. By contrast, the carbon nanofibers with a smaller diameter help to shorten the diffusion length for both Li‐ions and electrons. This effect can be regarded as a positive factor promoting the electrochemical performance of LIBs.^8^, ^10^, ^11^

The SEM images in Figure 2i–k show the surface morphology of the convex, indicating that the central part of the convex has a rough and bare surface. Theoretically, this appearance contributes to direct contact between the electrode material and copper substrate, which is considered to be an efficient strategy to ensure the electrical conductivity of the electrode.^43^ It is noteworthy that some scattered carbon nanofibers are also deposited on the surface of the convex due to the catalysis of the nanosized copper fins. Because of the lower catalytic activity of the nanosized copper fins, the carbon nanofibers on the surface of the convex are relatively dispersive and sparse so that the electrical conductivity of the convex shall not be seriously influenced. The solid structures of the samples in Figure 2d,h further demonstrate that the fibrous carbon products on the copper plate are carbon‐based nanofibers rather than nanotubes. In addition, the selected area electron diffraction (SAED) pattern in Figure 2l manifests that the internal structure of the carbon nanofibers is amorphous, which is believed to be a dominant factor beneficial to the electrochemical performances of the battery.^9^, ^50^ In contrast to the checkerboard‐like Cu@CNF current collector, the features of surface structure and composition of the complanate Cu current collector are greatly restricted (see Figure S1 in the Supporting Information). As a consequence, the performance of the battery based on this current collector must be partly limited.

**Figure 3:**
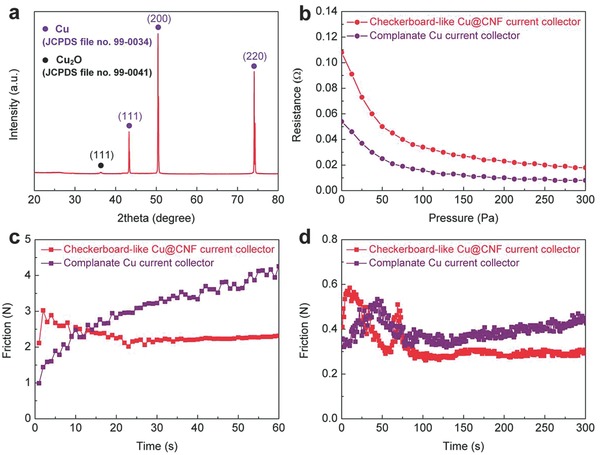
a) XRD pattern of the checkerboard‐like Cu@CNF current collector; b) relationship between the resistance and pressure in the cases of checkerboard‐like Cu@CNF and complanate Cu current collectors; c) point‐surface friction curves; and d) surface–surface friction curves.

The crystallographic details of the checkerboard‐like Cu@CNF current collector are characterized by X‐ray diffraction (XRD) test. As shown in the XRD patterns (**Figure** **3**a), two different phase structures are found. The diffraction peaks marked with purple symbols can be indexed to the copper crystal (JCPDS file no. 99‐0034), corresponding to the copper substrate of the current collector. The weak diffraction peak at 2θ value of 36.4°could be assigned to the cuprous oxide crystal (JCPDS file no. 99‐0041). This should be inevitably caused by the oxidation of the copper substrate in the air, owing to the high activity of the nanosized copper fins. It can be seen that the characteristic peak of the Cu_2_O is very weak, indicating a low content of the Cu_2_O impurity. Therefore, we can conclude that the Cu_2_O has little effect on the battery performance even the Cu_2_O cannot be avoided completely. In addition, there is no diffraction peak of carbon or graphite in the XRD pattern of the checkerboard‐like Cu@CNF current collector due to the amorphous structure of the carbon nanofibers, which is in good agreement with the TEM analysis. However, there is also no diffraction peak of nickel in the XRD pattern despite the current collector going through nickel plating. This is because the amorphous carbon nanofibers are deposited on the grooves and completely cover the nickel catalyst after the CVD process, thereby obstructing the recognition of crystallographic information for the nickel layer. Furthermore, the nickel plating of the current collector only lasts for a short time. Therefore, the crystallographic information of the nickel is much less obvious due to the low content of the nickel catalyst. During the assembly process of the CR2032 coin half‐cells, we applied a large pressure to their shells to improve the tightness. Therefore, it is necessary to disclose the relationship between the electrical conductivity of the electrodes and the external pressure.^51^ As shown in Figure 3b, the resistance of these electrodes decreases to a relatively stable level with the increase in external pressure. Under the effect of increasing pressure, the electrode and current collector become much closer so as to reduce the interfacial contact resistance. Furthermore, it is worth noting that the electrode based on the new current collector has a larger stable resistance (0.018 Ω) than that with a complanate pattern (0.008 Ω). The higher resistance might come from the additional resistance for the current collector due to the presence of carbon nanofibers.

To illuminate the effect of the graphical carbon nanofibers, the interfacial bonding strength between the electrode material and current collector was investigated through the method of mechanical friction (see the Experimental Section; Figure S2b,c, Supporting Information). It can be seen that the checkerboard‐like Cu@CNF current collector yields a much larger initial point‐surface friction than the complanate copper sample under the same condition, as shown in Figure 3c. This result suggests that the graphical carbon nanofibers and grooves contribute to increasing the surfaceness of the current collector. Due to the lubrication of the crush carbon nanofibers, the point‐surface friction for the checkerboard‐like Cu@CNF current collector gradually decreases to a relatively stable level. However, this is not the case for a complanate one. The surfaceness of current collector has a significant effect on the interfacial bonding strength between the electrode material and current collector. To validate this rationale, Figure 3d provides the surface–surface friction curves of the electrodes based on these two types of current collectors. We can find that the curve for the new current collector has a higher main peak (0.571 N) than that for the complanate sample (0.534 N). This result verifies the better interfacial bonding strength of the electrode based on the checkerboard‐like Cu@CNF current collector, which is consistent with the surfaceness test. Moreover, the secondary peak of the curve for the checkerboard‐like Cu@CNF current collector is caused by the graphical carbon nanofibers and grooves, which also agrees well with the curves in Figure 3c. Therefore, we can conclude that these special surface structures help to increase the interfacial bonding strength between the electrode material and current collector, and thereby enhance the electrochemical performance of the electrode.

**Figure 4:**
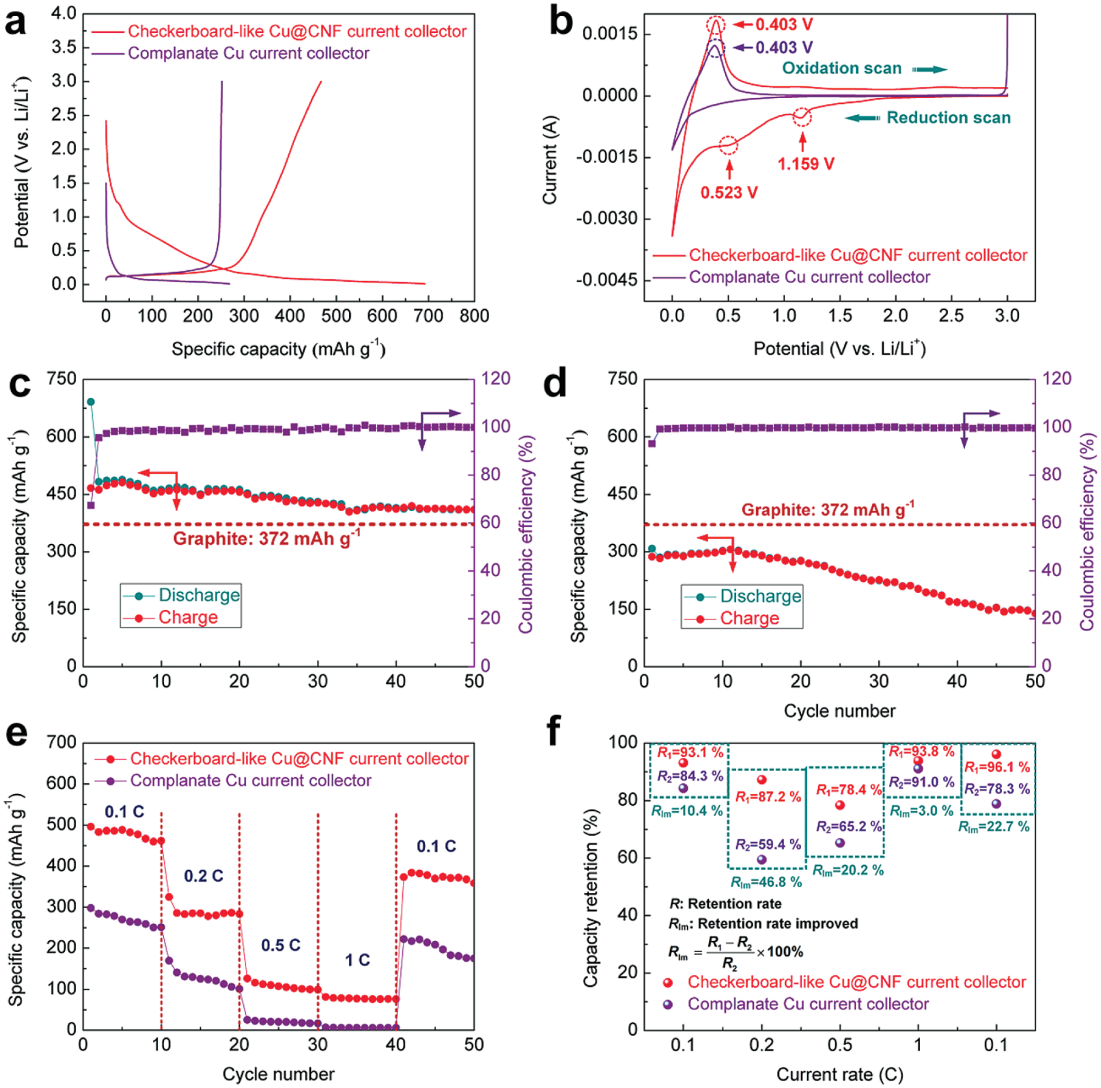
a) Initial voltage‐capacity plots for the as‐prepared batteries at a current rate of 0.1 C; b) initial CV curves for the batteries at a scan rate of 0.2 mV s^−1^; Discharge–charge performance of the batteries based on c) the checkerboard‐like Cu@CNF current collector and d) the complanate Cu current collector at a current rate of 0.1 C; e) rate capability and f) capacity retention of the batteries at different current rates.

To support the aforementioned opinion, the cycle performance and rate capability were investigated systematically, as shown in **Figure** **4**. It can be seen from Figure 4a that under the effects of the grooves and carbon nanofibers, the battery with the new current collector exhibits a much higher initial discharge voltage (2.42 V) and capacity (691.7 mAh g^−1^) than that with a complanate one (1.498 V and 267.9 mAh g^−1^, respectively). The extra capacity beyond the theoretical value of pure graphite (372 mAh g^−1^) should derive from the irreversible side reactions including the formation of the solid electrolyte interface (SEI) during the first discharge,^17^, ^43^, ^44^ as illustrated in Figure 1b. However, as shown in Figure S3b in the Supporting Information, these side reactions gradually disappear in the subsequent cycles since a stable SEI film has been formed on the surface of the electrode, obstructing the further consumption of the electrolyte. For a more in‐depth investigation, Figure 4b depicts the initial cyclic voltammetry (CV) curves for the electrodes (current collectors loaded with mesocarbon microbead (MCMB)) at a scan rate of 0.2 mV s^−1^. During the first reduction scan, there are two peaks observed at 1.159 and 0.523 V for the batteries with the checkerboard‐like Cu@CNF current collectors. According to the surface composition of this current collector, the distinct peak at 1.159 V is attributed to the reaction of lithium ions with residual oxygen‐containing functional groups, and the one at 0.523 V corresponds to the formation of SEI films on the amorphous carbon nanofibers.^50^ In contrast, these reaction peaks are not obvious for the electrode based on the complanate Cu current collector due to its monotonous surface composition. In addition, the batteries based on these current collectors show their reaction peaks at ≈0.403 V in the first oxidation scan. Analogously, this phenomenon can be ascribed to the delithiation reaction of the active materials. The reaction peaks in the CV curves agree well with the plateaus of the discharge–charge curves for the as‐prepared electrodes (see Figure 4a,b; Figure S3 in the Supporting Information). Moreover, after the first cycle, the voltage‐capacity plots and the CV curves for the electrodes both become more similar in shape (see Figure S3 in the Supporting Information), indicating that the reversibility of the electrode reactions increases. This can be also confirmed by the disappearance of the reduction peaks at 1.159 and 0.523 V in the CV curves for the electrodes based on the checkerboard‐like Cu@CNF current collectors in the subsequent cycles.

Figure 4c,d compares the discharge–charge performances of the batteries based on these two types of current collectors at a current rate of 0.1 C. It can be seen that the battery with the checkerboard‐like Cu@CNF current collector exhibits an excellent cyclic reversible capacity of 410.1 mAh g^−1^ with a capacity retention ratio of more than 84.9%, even after 50 cycles (see Figure 4c). By contrast, the battery with a complanate current collector only retains its discharge capacity of 138.8 mAh g^−1^ and capacity retention ratio of 48.7% under the same operating conditions (see Figure 4d). This result demonstrates that the battery using the new current collector yields a much higher reversible capacity beyond the theoretical capacity of carbonaceous materials and better cycling stability than the complanate Cu current collector. The excellent cycle performance relates closely to the surface structure of the checkerboard‐like Cu@CNF current collectors. Specifically, as illustrated in Figure 1b, the graphical carbon nanofibers are more able to provide efficient conductive paths for the MCMB active materials and open up more reaction sites for fast electron transport. Hence, the reversible capacity and cycling stability of the battery can be significantly intensified. Furthermore, these amorphous carbon nanofibers keep twining around the MCMB active materials during the lithiation and delithiation processes. As a result, the volume variation of the active materials can be significantly restrained, thereby extending the cycle life of the electrode. Moreover, the patterned grooves contribute to increasing the surface area of the current collector. With increasing the surface area, the effective contact points between the electrode material and current collector can remarkably be increased. Accordingly, the electrical conductivity and bonding strength of the electrodes are significantly improved. Besides, the volume variation of the active material stored in the grooves is also partly restrained owing to the special structure of the patterned grooves. Consequently, the cycling stability and cycle life of the electrodes are prominently enhanced. By the comparison of Figure 4c,d, we can see that the capacity of the battery with the checkerboard‐like current collector becomes more stable from the 40th cycle while the capacity of the battery with the complanate one tends to decrease. Therefore, we can conclude that the battery with the checkerboard‐like current collector yields a better cycle performance than that with the complanate one in the subsequent cycles.

Figure 4e shows the rate capability of the as‐prepared batteries. It can be seen that the batteries with the checkerboard‐like Cu@CNF current collectors still maintain their discharge capacities of 461.8, 238.2, 98.9, 76, and 358.4 mAh g^−1^ after ten cycles at the current rates of 0.1 C, 0.2 C, 0.5 C, 1 C, and 0.1 C, respectively. The enhanced rate capability can be ascribed to the superior electrical conductivity of the current collector and less‐severe volume variation of the active materials restrained by the grooves and carbon nanofibers. By comparison, the batteries with complanate Cu current collectors only present discharge capacities of 250.9, 100.8, 16.5, 6.1, and 175.1 mAh g^−1^ after ten cycles at the current rates of 0.1 C, 0.2 C, 0.5 C, 1 C, and 0.1 C, respectively, owing to poor electrical conductivity of the electrodes and the severe volume variation of active materials.

More specifically, Figure 4f depicts the capacity retention of the as‐prepared batteries at different current rates. The detailed values are listed in Table S1 in the Supporting Information. It is evident that the batteries with the checkerboard‐like Cu@CNF current collectors keep much higher capacity retention (*R*_1_: 93.1%, 87.2%, 78.4%, 93.8%, and 96.1% at 0.1 C, 0.2 C, 0.5 C, 1 C, and 0.1 C, respectively) than the batteries with complanate current collectors (*R*_2_: 84.3%, 59.4%, 65.2%, 91.0%, and 78.3% at 0.1 C, 0.2 C, 0.5 C, 1 C, and 0.1 C, respectively) at different current rates. Importantly, the capacity retention rates are increased by 10.4%, 46.8%, 20.2%, 3.0%, and 22.7% at the current rates of 0.1 C, 0.2 C, 0.5 C, 1 C, and 0.1 C, respectively. Furthermore, the battery with a checkerboard‐like Cu@CNF current collector still delivers a stable capacity of about 358.4 mAh g^−1^ with a capacity retention rate of more than 77% at a current density of 0.1 C after each rate cycle. Whereas the use of a complanate current collector retains only 175 mAh g^−1^ with a capacity retention rate of 69.7%. These results demonstrate that the battery with a checkerboard‐like Cu@CNF current collector yields a higher rate performance than the complanate current collector. We believe that this remarkable improvement benefits significantly from the synergistic effect of the grooves and carbon nanofibers on the surface of the current collector.

**Figure 5:**
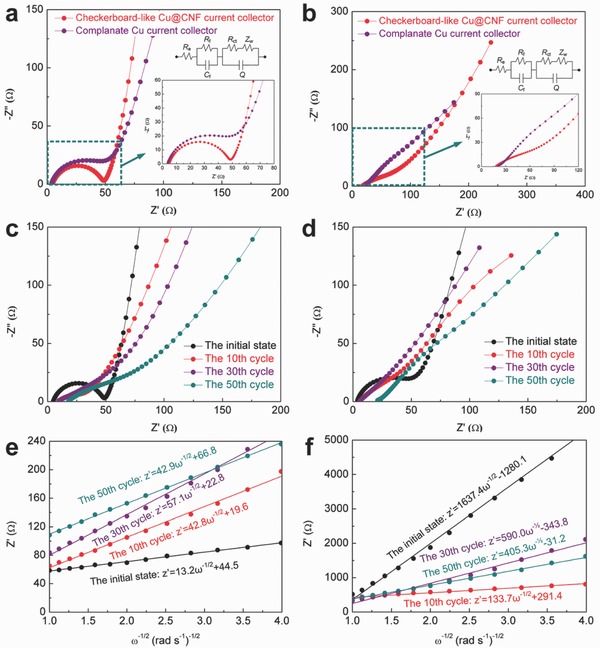
Electrochemical impedance curves for the as‐prepared batteries a) at the initial state and b) after 50 cycles. Inset in (a) and (b): magnified images of the selected regions and the equivalent circuit for the batteries; Electrochemical impedance curves for the batteries based on c) the checkerboard‐like Cu@CNF current collector and d) the complanate Cu current collector after different cycles; The relationship of the imaginary resistance with the inverse square root of the angular speed for the batteries based on e) the checkerboard‐like Cu@CNF current collector and f) the complanate Cu current collector after different cycles.

To clarify the essential differences of the electrochemical behaviors of the prepared current collectors, **Figure** **5**a,b illustrates the electrochemical impedance spectroscopy (EIS) curves of the electrodes at the initial state and after 50 cycles, respectively. We can roughly find that the battery with the checkerboard‐like Cu@CNF current collector has a smaller impedance than the complanate current collector. This result may be also attributed to the combined effects of the grooves and carbon nanofibers on the surface of the current collector. To validate this hypothesis, the impedance curves are calculated by an equivalent circuit inserted in Figure 5a,b, and the detailed impedance parameters are listed in Table S2 in the Supporting Information. The calculated situations of the impedance curves ensure the accuracy of the equivalent circuit (see Figure S4 in the Supporting Information). Here, the parameters *R*_e_, *R*_f_, *R*_ct_, and *Z*_w_ correspond to the Ohmic resistance of the electrolyte and electrode in a battery, the Li‐ion migration resistance in the SEI film, the charge transfer resistance of corresponding electrochemical reactions, and the Warburg impedance related to Li‐ion diffusion, respectively. *C*_f_ and *Q* in the equivalent circuit are related to the SEI film capacitance and double‐layer capacitance of the working electrodes, respectively.^52^ At the initial state, the battery using a checkerboard‐like Cu@CNF current collector has an approximately equal value of *R*_e_ (4.803 Ω) with that with a complanate pattern (4.672 Ω). This indicates that both batteries have identical internal environment (see Table S2 in the Supporting Information). However, we can identify a great difference in *R*_f_ and *R*_ct_ between the two batteries. Specifically, the battery with the new current collector produces a much larger *R*_f_ (6.708 Ω) and smaller *R*_ct_ (43.06 Ω) compared with that using a complanate current collector (1.832 and 57.42 Ω, respectively). According to the surface structure of the checkerboard‐like Cu@CNF current collector, a higher value of *R*_f_ is associated with the additional SEI resistance on the carbon nanofibers, and a smaller *R*_ct_ relates closely to high electrical conductivity of the carbon nanofibers. It is noteworthy that *R*_e_ and *R*_ct_ of the both batteries are greatly increased after 50 cycles, resulting from the different‐level pulverization of the electrodes. However, owing to the effect of the grooves and carbon nanofibers, the battery with the new current collector presents a much smaller *R*_e_ and *R*_ct_ than that based on a complanate pattern. As a result, the total resistance (*R*_∑_) of the newly structured battery is remarkably reduced, which highlights the excellent electrical conductivity of the battery. Moreover, the essentially constant *R*_f_ of both batteries reveals that the SEI films on the electrodes become more stable. To clarify the different electrochemical behaviors of the two batteries, Figure 5c,d demonstrates the impedance curves of the electrodes after different cycles at a current rate of 0.1 C. It can be seen that the electrochemical impedance of the two batteries increases with the increase in cycle number due to the pulverization of the electrode materials. Besides, the impedance related to the complanate current collector dramatically change with increased cycle number, whereas the curve for the checkerboard‐like pattern is relatively stable. This phenomenon may significantly depend on the morphology of the as‐prepared current collectors.

The diffusion coefficient of LIBs is considered as one of the most important parameters to evaluate the performance of the electrodes. As reported by the previous literatures,^52^, ^53^ the diffusion coefficient of lithium ions (*D*_Li_^+^) in the electrodes can be calculated from a linear relationship between *Z*′ and ω^−1/2^, as described by the following model

(1) $$D_{{\mathrm{Li}}^{+}}\;=\;\frac{R^{2}T^{2}}{2A^{2}n^{4}F^{4}C^{2}\sigma^{2}}$$

(2) $$Z_{w}\;=\;R_{D}\;+\;R_{L}\;+\;\sigma \omega^{-1/2}$$

Here, *R* is the gas constant, *T* is the test absolute temperature in test environment, *A* is the surface area of the working electrode, *n* is the number of electrons present in the electronic transfer reaction, *F* is Faraday constant, *C* is the concentration of lithium ion in the working electrode, and ω is the angular frequency. Moreover, the value of σ can be acquired from the plots shown in Figure 5e,f, which represents the slope of *Z*′ against ω^−1/2^. It is obvious that the battery with a checkerboard‐like Cu@CNF current collector leads to a much smaller σ than the complanate pattern after different cycles. According to the above equations, this result indicates that using the new current collector results in a relatively larger diffusion coefficient of lithium ions under the same conditions. Generally, after the first cycle, the *D*_Li_^+^ for the battery with a common current collector will decrease with the increase in cycle number (see Figure 5f). However, as shown in Figure 5e, the value of *D*_Li_^+^ for the battery with the new current collector decreases first and then increases with the augment of cycle number. This phenomenon indicates that the checkerboard‐like Cu@CNF current collector has a much better ability to alleviate the volume variation and pulverization of the electrode material layer, which further highlights the superiority of its practical application in LIBs.

## 3 Conclusion

In summary, we have developed a simple and cost‐effective process for nickel plating and fabrication of 3D checkerboard‐like Cu@CNF composite current collectors for LIBs. The patterned grooves and amorphous carbon nanofibers not only increase the contact area between the active electrode material and current collector, but also help restrain the volume variation of the electrode material during cycling. Due to the combined effect of the grooves and the carbon nanofibers, a battery based on this current collector exhibits significantly enhanced reversible capacity, rate capability, and cycle life. This novel composite current collector is applicable to other high‐performance electrochemical cells as well.

## 4 Experimental Section

*Preparation of Checkerboard‐Like Cu@CNF Current Collectors*: Carbon nanofibers were regularly deposited on pretreated copper plates by CVD using a quartz tube furnace (FWL(ZK)‐08/70/3, China). For pretreatment, the two surfaces of the common copper plate with a thickness of 1 mm and a diameter of 15 mm were polished with fine sandpapers to remove the contaminants. One surface (denoted as surface A) of the copper plate was then covered with an anticorrosion photosensitive film and the counter surface was entirely naked. Next, the designed checkerboard‐like pattern was exposed to the surface A with an ultraviolet exposure machine and treated in the developer solution. After this, the pretreated copper plate was placed in the (NH_4_)_2_S_2_O_8_ solution at a concentration of 0.2 g mL^−1^ with the surface A facing upward and etched for 15 min. When the etching process completed, the copper plate was transferred to the electroless nickel plating solution (see Table S3 in the Supporting Information) for nickel plating (see Figure S5 in the Supporting Information). The common nickel plate was first placed in the plating solution at 80 °C. After its surface bubbled, the as‐prepared copper plate was transferred to the plating solution and contacted closely with the nickel plate for 10 s. Subsequently, the nickel plate was removed out and the copper plate was kept in the plating solution for another 5 min to finish the nickel plating process. Finally, the copper plate was treated in the absolute alcohol with a purity of 99.7% to strip the anticorrosion photosensitive film from its surfaces. To activate the nickel catalyst, the copper plate was dipped in the catalyst activating solution (see Table S3 in the Supporting Information) for 30 min. The copper plate was then washed with deionized water and completely dried in an oven at 60 °C.

After drying, the copper plate was loaded into the CVD chamber with its surface A facing upward. Then the CVD chamber was pumped down to 10^−2^ torr and backfilled with flowing argon to atmospheric pressure. Afterward, the copper plate was heated up to 650 °C in the mixture of argon and hydrogen gases at a flow rate of 50 and 10 sccm respectively, following which the acetylene at a flow rate of 40 sccm was pumped into the CVD chamber. Finally, the copper plate was annealed at 650 °C for 20 min, where the checkerboard‐like Cu@CNF current collector was formed. For comparison, Cu current collectors with complanate structures were also prepared.

*Structural Characterization*: The crystallographic details of the Cu@CNF current collector were characterized in the 2θ range of 20°–80° by using X‐ray diffraction spectrometry (Bruker, Germany). The surface morphologies of the current collector were examined by a field emission scanning electron microscope (Zeiss Merlin, Germany). Besides, the internal structure of the samples was observed on a transmission electron microscope (JEOL 2100 F, Japan) at the accelerating voltage of 200 kV.

*Electrical Conductivity and Mechanical Tests*: Before testing, the electrode was prepared by coating electrode material slurry evenly on the surface of the current collector and completely dried. The slurry was composed of four types of materials: carboxymethyl cellulose, styrene butadiene rubber, Super‐P carbon, and MCMB graphite powder with a weight ratio of 2:2:3:93. The mass loading of the MCMB graphite powder is ≈7.5 mg for the both batteries. The thickness of the MCMB layer is about 78.63 µm and 77.54 µm for the battery with the checkerboard‐like current collector and the complanate one, respectively (see Figure S6a,b in the Supporting Information). The electrical conductivity of the electrode was tested using the voltammetry method (see Figure S2a in the Supporting Information). Since the surface profile of a current collector directly affects the physical strength of the electrode, the surfaceness of a current collector and the interfacial bonding strength between the electrode material layer and current collector were evaluated using a precision friction and wear tester (MPX‐1G, China), as shown in Figure S2b,c in the Supporting Information, respectively.

*Electrochemical Measurements*: The electrochemical performance of the electrode was investigated by using CR2032 coin half‐cells assembled in an Ar‐filled glove box (O_2_, H_2_O levels maintained at <0.1 ppm) with a lithium foil as the reference electrode and Celgard 2325 as the separator (see Figure S2d–f in the Supporting Information). The electrolyte is made from LiPF_6_ (organic solvent: EC+DMC+DEC, 1:1:1 by volume). The cycle performances of the cells were evaluated on a battery testing platform (CT2001A, LANHE, China) through repeated discharge–charge processes over a voltage range of 0.02–3 V versus Li/Li^+^ at a room temperature. The CV and EIS measurements were conducted on a electrochemical work station (PGSTAT302N, Autolab, Switzerland). The CV tests were carried out at a scan rate of 0.2 mV s^−1^ and the impedance spectra were recorded by prescribing an amplitude of 5 mV in the working frequency range of 10^−2^–10^5^ Hz.

## Conflict of Interest

The authors declare no conflict of interest.

## Supporting information

Supplementary
