# Supplementary material for: From Checkerboard‐Like Sand Barriers to 3D Cu@CNF Composite Current Collectors for High‐Performance Batteries
Source: Adv Sci (Weinh). 2018 Mar 27;5(7):1800031. doi: 10.1002/advs.201800031 (PMC6051219; doi:10.1002/advs.201800031)
Supplement: Supplementary file 1 — Supplementary [file ADVS-5-1800031-s001.pdf]

Copyright WILEY-VCH Verlag GmbH & Co. KGaA, 69469 Weinheim, Germany, 2018.

ADVANCED SCIENCE
Open Access

Supporting Information

for *Adv. Sci.,* DOI: 10.1002/advs.201800031

From Checkerboard-Like Sand Barriers to 3D Cu@CNF Composite Current Collectors for High-Performance Batteries

Jian Luo, Wei Yuan, Shimin Huang, Bote Zhao, Yu Chen, Meilin Liu,* and Yong Tang*

Copyright WILEY-VCH Verlag GmbH & Co. KGaA, 69469 Weinheim, Germany, 2018.

Supporting Information

From Checkerboard-Like Sand Barriers to Three-Dimensional Cu@CNF Composite Current Collectors for High-Performance Batteries

Jian Luo, Wei Yuan, Shimin Huang, Bote Zhao, Yu Chen, Meilin Liu,* and Yong Tang*

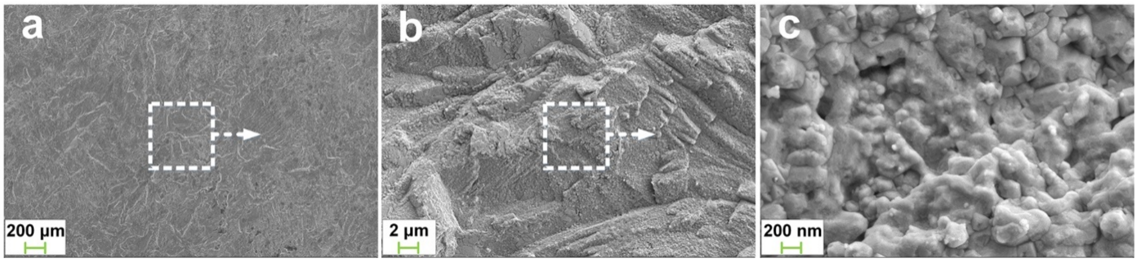

Figure S1. a) SEM image and b, c) enlarged SEM images of the complanate Cu current collector. The monotonous surface morphology of the complanate Cu current collector will severely limit the electrical conductivity and strength of the electrode, and thereby degrade the electrochemical performance of batteries.

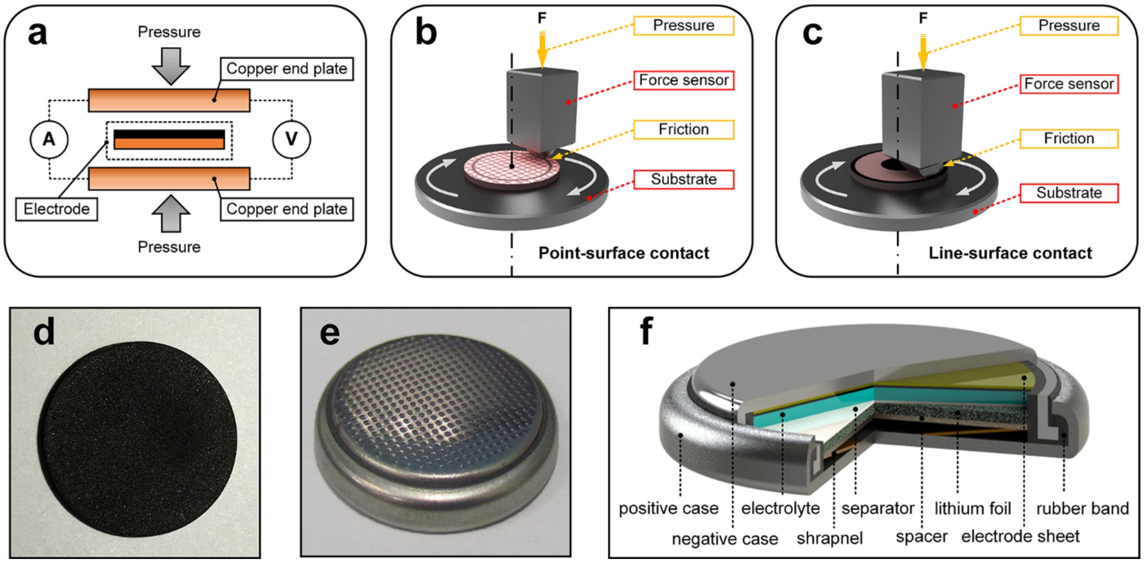

Figure S2. a) Schematic diagram of the electrical conductivity test for the as-prepared electrode. During the testing process, external pressures with gradient of 12.5 N are applied on the copper end plates. The voltages (U) and currents (I) under different pressure conditions can be acquired by using multimeter and DC power source, and ohmic resistances (R) of the electrodes can be calculated by the following equation: $R = U / I$. Schematic illustrations of b) the point-surface friction test and c) surface-surface friction test. For a reasonable test, a constant pressure (F) should be loaded on the force sensor. The friction coefficient (μ) can be obtained by the force sensor and the friction (F_μ) can be calculated by the equation: $F_\mu = \mu F$. The rotate speed of the substrate is 100 r min^{-1}. Morphological images of d) the prepared electrode, e) CR2032 coin half-cell and f) structure of the CR2032 coin half-cell.

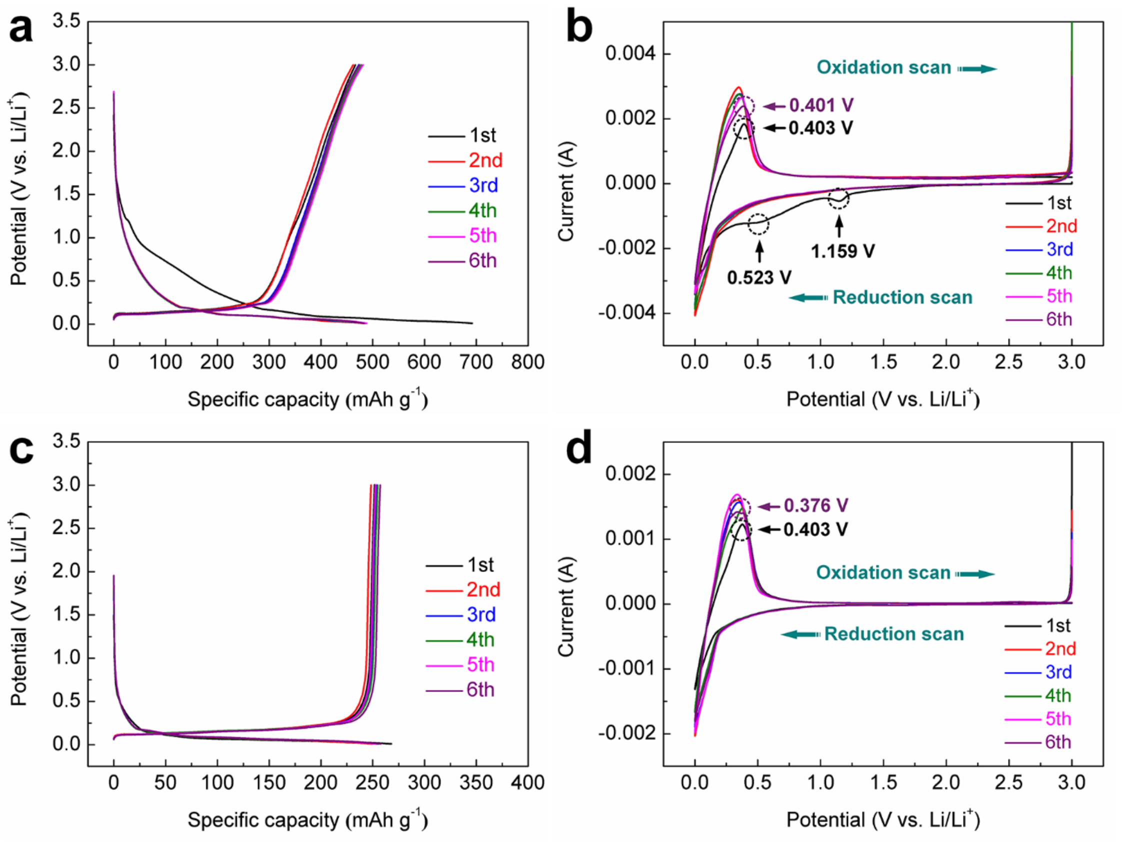

Figure S3. a) Voltage-capacity plots and b) CV curves for the first 6 cycles of the batteries based on the checkerboard-like Cu@CNF current collector at the current rate of 0.1 C; c) Voltage-capacity plots and d) CV curves for the first 6 cycles of the batteries based on the complanate Cu current collector at the current rate of 0.1 C. The more similar shape of these curves and the slightly declined voltage of the oxidation peak reveal the more reversible electrode reactions in the as-prepared batteries.

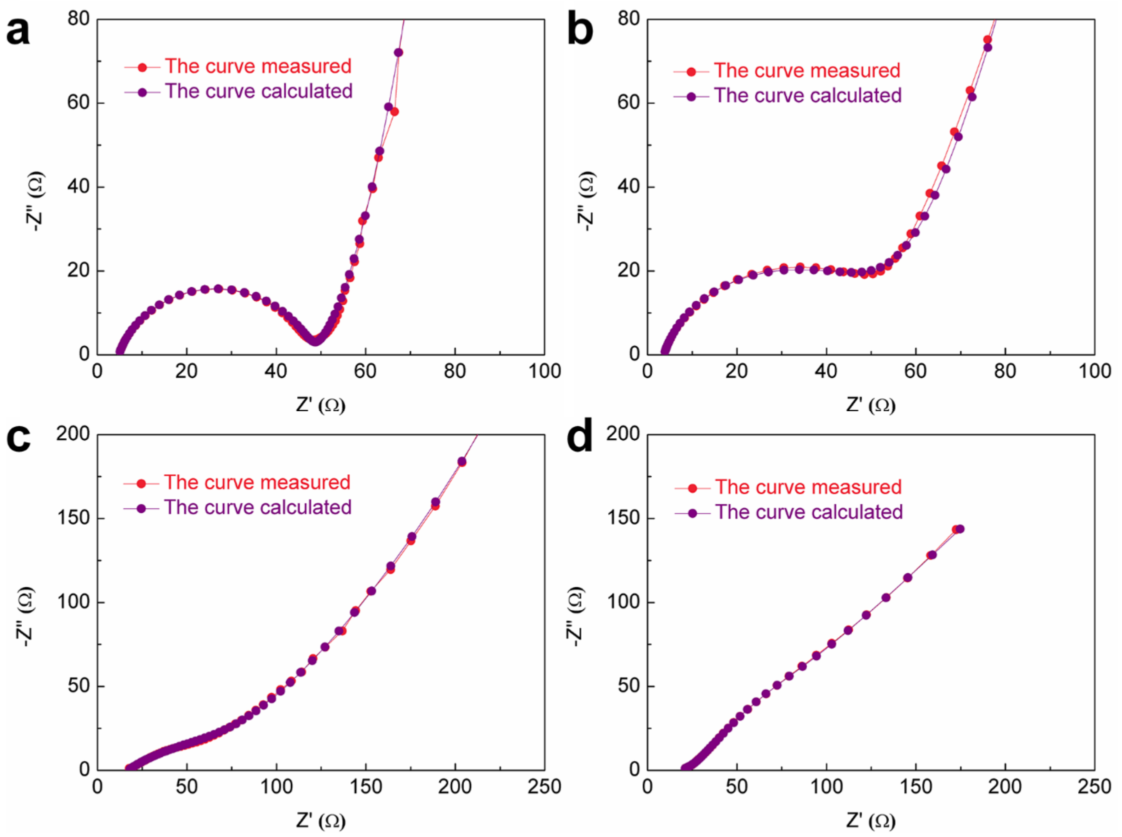

Figure S4. Calculated situations of the impedance curves for the batteries based on a, c) the checkerboard-like Cu@CNF current collector and b, d) the complanate Cu current collector at the initial state and after 50 cycles, respectively. Obviously, the curves measured are in good agreement with the curves calculated, which testifies the accuracy of the special equivalent circuit.

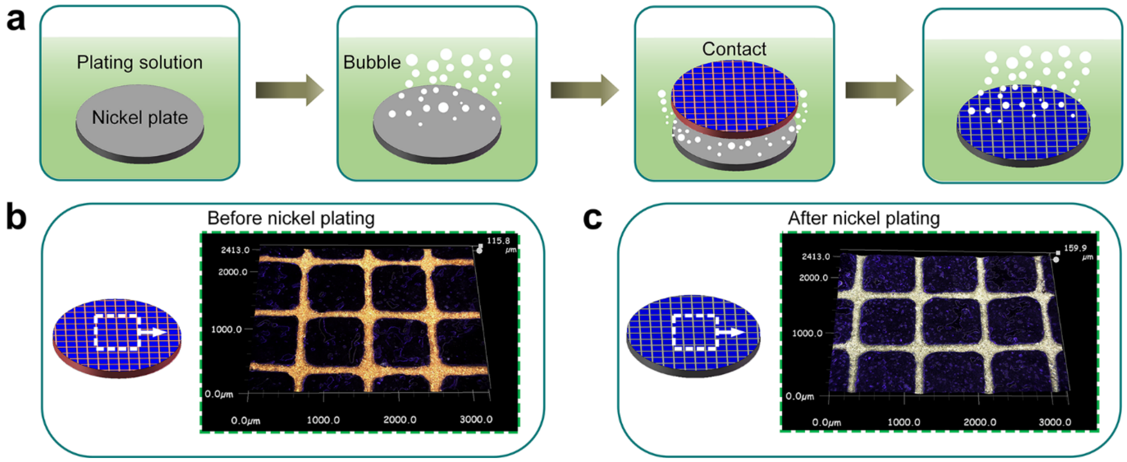

Figure S5. a) Schematic diagram of nickel plating on the surface of the etched copper plate. This novel nickel plating method completely avoids the sensitization and activation procedures in the traditional electroless nickel plating process. Conveniently, we can achieve nickel plating on conductive samples by simply contacting the samples with nickel plates in the electroless nickel plating solution. b) Digital microscope image of the etched copper plate before nickel plating process. c) Digital microscope image of the etched copper plate after nickel plating process. The silvery grooves on the surface of the sample shown in c) indicate that the nickel layer has been successfully plated in the grooves.

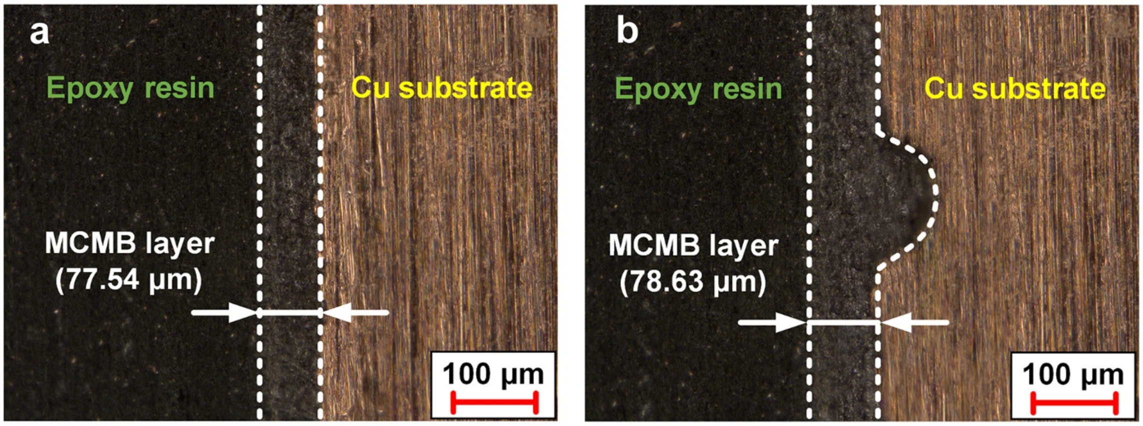

Figure S6. Digital microscope images of the cross sections for the electrodes with a) the checkerboard-like current collector and b) the complanate one. The epoxy resin in the images is used to prevent the MCMB layer shedding from the Cu substrate while polishing the cross sections of the electrodes.

Table S1. Capacity retention rate of the as-prepared batteries between the first and tenth cycles at different current rates.

Samples	C_1 [c)]	C_{10} [c)]	R [c)]	C_1 [d)]	C_{10} [d)]	R [d)]	C_1 [e)]	C_{10} [e)]	R [e)]	C_1 [f)]	C_{10} [f)]	R [f)]	C_1 [g)]	C_{10} [g)]	R [g)]
# 1[a)]	496.0	461.8	93.1	324.6	283.2	87.2	126.2	98.9	78.4	81.0	76.0	93.8	372.9	358.4	96.1
# 2[b)]	297.8	250.9	84.3	169.6	100.8	59.4	25.3	16.5	65.2	6.7	6.1	91.0	222.1	173.9	78.3

[a)]Battery based on the checkerboard-like Cu@CNF current collector; [b)]Battery based on the complanate Cu current collector; [c)]At the current rate of 0.1 C; [d)]At the current rate of 0.2 C; [e)]At the current rate of 1 C; [f)]At the current rate of 2 C; [g)]At the current rate of 0.1 C.

C_1: Specific capacity of the battery in the first cycle at a particular current rate.

C_{10}: Specific capacity of the battery in the tenth cycle at a particular current rate.

R: Capacity retention rate of the battery at a particular current rate.

Table S2. Impedance parameters derived using equivalent circuit model for the batteries based on the two types of current collectors at the initial state and after 50 cycles.

Samples		R_e (Ω)	R_f (Ω)	R_{ct} (Ω)	R_Σ (Ω)
# 1[a]	State 1[c]	4.803	6.708	43.06	54.571
	State 2[d]	17.12	5.506	89.14	111.766
# 2[b]	State 1[c]	4.672	1.832	57.42	63.924
	State 2[d]	19.61	1.292	158.69	179.592

[a]Battery based on the checkerboard-like Cu@CNF current collector; [b]Battery based on the complanate Cu current collector; [c]At the initial state; [d]After 50 cycles.

Table S3. Formulations of the electroless nickel plating solution and catalyst activating solution.

Chemical	Formula	Concentration
Sodium hypophosphite[a)]	$NaH_2PO_2 \cdot H_2O$	20 g L^{-1}
Nickel sulfate[a)]	$NiSO_4 \cdot 6H_2O$	40 mL L^{-1}
Sodium citrate[a)]	$NaC_6H_5O_7 \cdot 2H_2O$	100 g L^{-1}
Ammonium chloride[a)]	NH_4Cl	50 mL L^{-1}
Hydrochloric acid[b)]	HCl	40 mL L^{-1}
Copper chloride[b)]	$CuCl_2 \cdot 2H_2O$	20 g L^{-1}

[a)]Formulations of the electroless nickel plating solution; [b)]Formulations of the catalyst activating solution.
